# Maternal Serum Melatonin Increases During Pregnancy and Falls Immediately After Delivery Implicating the Placenta as a Major Source of Melatonin

**DOI:** 10.3389/fendo.2020.623038

**Published:** 2021-02-18

**Authors:** Haroon Ejaz, Juliana K. Figaro, Andrea M. F. Woolner, Bensita M. V. Thottakam, Helen F. Galley

**Affiliations:** ^1^ Institute of Medical Science, University of Aberdeen, Aberdeen, United Kingdom; ^2^ Institute of Applied Health Science, University of Aberdeen, Aberdeen, United Kingdom

**Keywords:** melatonin, melatonin (6-sulfatoxymelatonin), pregnancy, placenta, antioxidant

## Abstract

Melatonin is a neuroendocrine hormone which regulates circadian rhythm and is also an antioxidant. The role of melatonin in pregnancy is emerging. The enzymes needed for endogenous synthesis of melatonin have been identified in the placenta, although the contribution to circulating maternal melatonin in normal pregnancy is unclear. This work aimed to determine serum levels of melatonin and its major metabolite 6-hydroxymelatonin sulfate (6-OHMS) in normal pregnant women during each trimester of pregnancy, and immediately after delivery. Blood samples were obtained from a cohort of healthy pregnant women during each trimester of pregnancy (n = 26), from women scheduled for elective Cesarean section (CS) before and after delivery (n = 15), along with placental samples, and from healthy non-pregnant women as controls (n = 30). Melatonin and its major metabolite, 6-OHMS, were measured using enzyme immunoassay. Levels of serum melatonin were significantly higher during pregnancy than in non-pregnant women (P = 0.025) and increased throughout pregnancy (P < 0.0001). In women undergoing CS, serum melatonin decreased markedly 24 h after delivery (P = 0.0013). Similar results were seen for serum levels of 6-OHMS, and placental tissue 6-OHMS levels correlated with week of gestation at delivery (p = 0.018). In summary, maternal melatonin production is higher in pregnant than in non-pregnant women, increases significantly during pregnancy with highest levels in the third trimester, and decreases abruptly after delivery. These results suggest that the placenta is a major source of melatonin and supports a physiological role for melatonin in pregnancy.

## Introduction

Oxidative stress is recognized as a potential pathophysiological mechanism for placental disease such as pre-eclampsia (1). Melatonin, as well as having a role in circadian rhythm regulation, is also a powerful antioxidant with anti-inflammatory actions (2, 3). Research has shown that melatonin has a role in pregnancy, specifically in placental function and physiology (4, 5). Earlier studies have reported higher melatonin levels in groups of pregnant compared to non-pregnant women (6–8). In addition, lower melatonin levels were seen in women with severe pre-eclampsia, associated with the severity of pre-eclampsia as shown in a meta-analysis (9).

Endogenous melatonin is synthesized from tryptophan *via* serotonin under the control of *N*-acetylserotonin-*O*-methyl-transferase, and aralkylamine-*N*-acetyl-transferase. Most endogenous melatonin production takes place in the pineal gland, regulated by light levels. However, e*x vivo* studies using human placental and ovary tissue report melatonin synthesizing enzymes capable of converting serotonin to melatonin (10–12). Such non-pineal sources are not regulated by light and therefore do not display circadian variation (13–15). However systematic assessment of endogenous circulating melatonin metabolism as pregnancy progresses, and identification of the placenta as the source of melatonin in pregnancy has not been reported. The aim of this study was to investigate circulating levels of melatonin and its major bioactive metabolite, 6-hydroxymelatonin sulfate (6-OHMS) during normal singleton pregnancy, compared to healthy non-pregnant women of child bearing age, and the contribution of the placenta.

## Materials and Methods

### Subjects and Study Design

The work presented here is from two linked prospective studies: in MEL-P we recruited healthy pregnant women carrying a single baby, plus healthy non-pregnant women of childbearing age. We then went on to recruit a second cohort of healthy pregnant women, also with singleton pregnancies, who were scheduled for elective Cesarean section (CS). This study was called MEL-P2. Both studies were prospectively registered at ClinicalTrials.gov (NCT03014245 and NCT03609086 respectively) and were undertaken at Aberdeen Maternity Hospital, NHS Grampian, Aberdeen, UK. The trials received research ethics approval and written informed consent was obtained from all participants.

#### Subjects

For both groups of pregnant women, women were excluded if they were under 16 or over 45 years, were carrying more than one baby or had a pre-pregnancy diabetes or hypertension, chronic kidney disease, or any known autoimmune disorder. For Study 1 (MEL-P) women with viable singleton pregnancies were recruited after their first trimester antenatal ultrasound scan at Aberdeen Maternity Hospital. Posters were placed in the ultrasound department and following their scan, eligible women were initially screened by antenatal clinical staff for eligibility and then asked if they wished to speak to a researcher about the study. Subjects who had abnormalities identified at the scan were excluded. After written informed consent, morning venous blood samples were obtained at 08:00–12:00 during each trimester (11–12 weeks, 19–21 weeks, and 27–29 weeks gestation) and women were followed up until delivery. To recruit healthy non-pregnant women of childbearing age, emails were sent from a third party to University and maternity staff email lists inviting non-pregnant women up to the age of 45 years to respond if they were interested in taking part. Anyone with any chronic health condition and/or was taking medication was excluded. After written informed consent, a single morning blood venous blood sample was obtained at 08:00–12:00 from these control subjects.

For study 2 (MEL-P2), pregnant women aged 16–45 years old with singleton pregnancies who were scheduled for elective CS were recruited. This study was designed to facilitate measurement of melatonin shortly before and immediately after delivery and to obtain placental tissue. After initial screening for eligibility by their midwife or doctor, women attending routine CS pre-assessment clinics were invited to speak with the researcher if they were interested in participating. After written informed consent, maternal morning blood samples were obtained at 08:00–12:00 between 3 and 6 days prior to elective CS and again 24 h after delivery and placental tissue was obtained within 15 min of delivery.

After delivery, gestational age at parturition, development of preterm labor, gestational diabetes, pregnancy-induced hypertension or pre-eclampsia were recorded for all pregnant participants.

#### Methods

Blood samples were collected into 5 ml clot activator-serum separator vacutainers, allowed to clot and then centrifuged within 2 h of collection, at 1,300 *g* for 10 min at room temperature. Serum was stored at −80°C until assay. Placental samples (1 cm^3^) were obtained from cotyledons that contained the cytotrophoblast and syncytiotrophoblast, from two diametrically opposed locations halfway between the center and the placental border and through the entire depth of the organ. Placental tissue samples were washed twice in Dulbecco’s phosphate-buffered saline (PBS, Gibco, Paisley, UK) to remove excess blood and snap frozen using a non-contact procedure (FlashFREEZE, Milestone Srl, Italy) where cryovials containing the samples were immersed in 1-methoxyheptafluoropropane (Novec 7000 Engineered Fluid, 3M, St. Paul, MN, USA), to achieve −80°C within 30 s. Frozen tissue was pulverized using a mortar and pestle under liquid nitrogen and the resulting powder (200 mg) was mixed with 2 ml PBS and pressed through a 100 mm strainer. Two cycles of freeze-thawing ensured rupture of cell membranes and homogenates were then centrifuged at 5,000 *g* for 5 min at 4°C prior to analysis.

Commercially available competitive enzyme immunoassays were used to measure melatonin (Wuhan Fine Biotech, Wuhan, China) and 6-OHMS (Abbexa Ltd., Cambridge, UK) after dilution, according to the manufacturer’s instructions. The between-plate precision of these assays in our hands was 8.9% for melatonin and 10.8% for 6-OHMS (coefficient of variation, n = 6). The protein content of tissue homogenates was measured using the Bradford technique with Coomassie Brilliant Blue dye (16) using bovine serum albumin as calibration standard (ThermoScientific, Rockford, IL, USA). All assays were undertaken in duplicate.

### Statistical Analyses

Data were not normally distributed and are presented as median, interquartile and full range. Statistical analysis was undertaken using Analyse-It statistical add-in for Microsoft Excel using Friedman test and Wilcoxon/Mann Whitney tests as appropriate. Spearman’s correlation was used to assess the relationship between gestational age and placental 6-OHMS content. A p value less than 0.05 was accepted as statistically significant.

## Results

### Participant Characteristics

**Figure 1:**
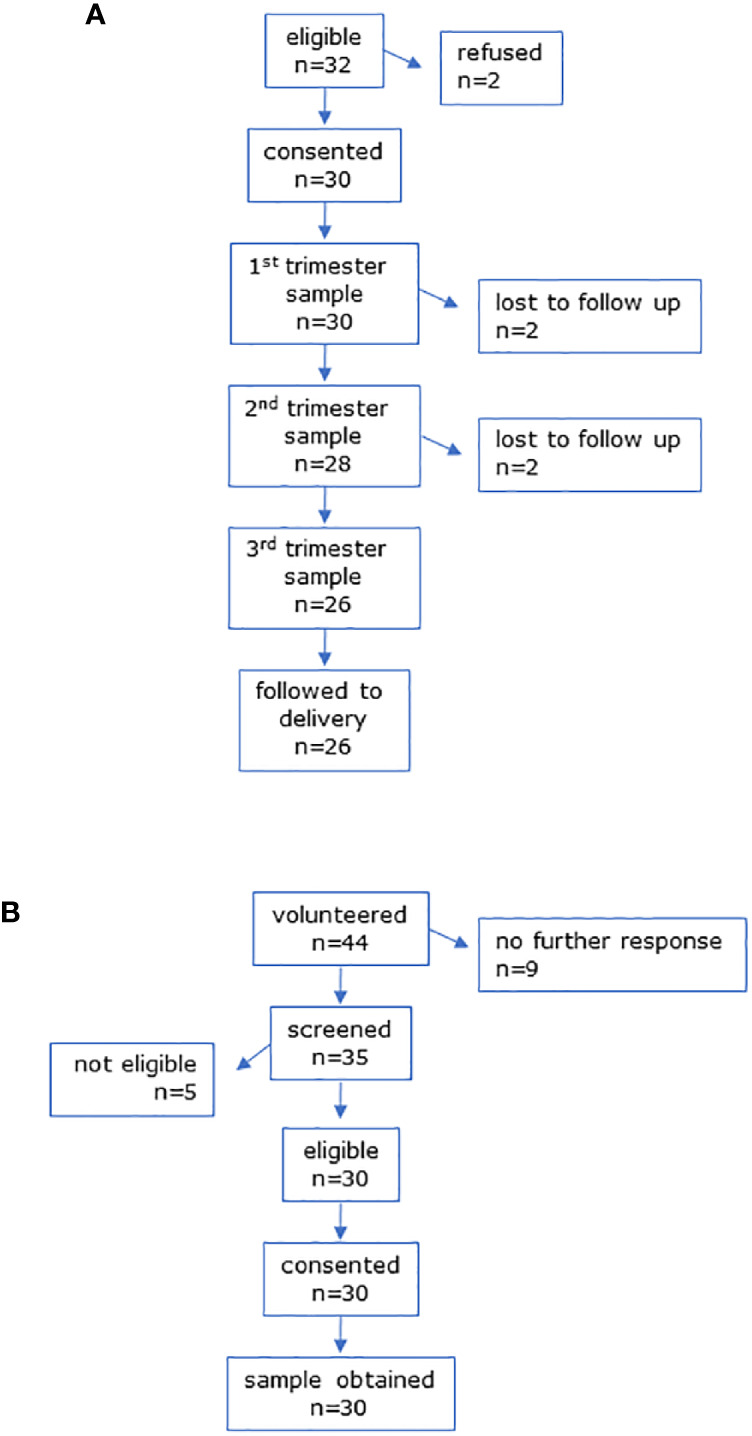
CONSORT diagrams for **(A)** MEL-P pregnant participants and **(B)** MEL-P Non-pregnant healthy control subjects.

**Figure 2:**
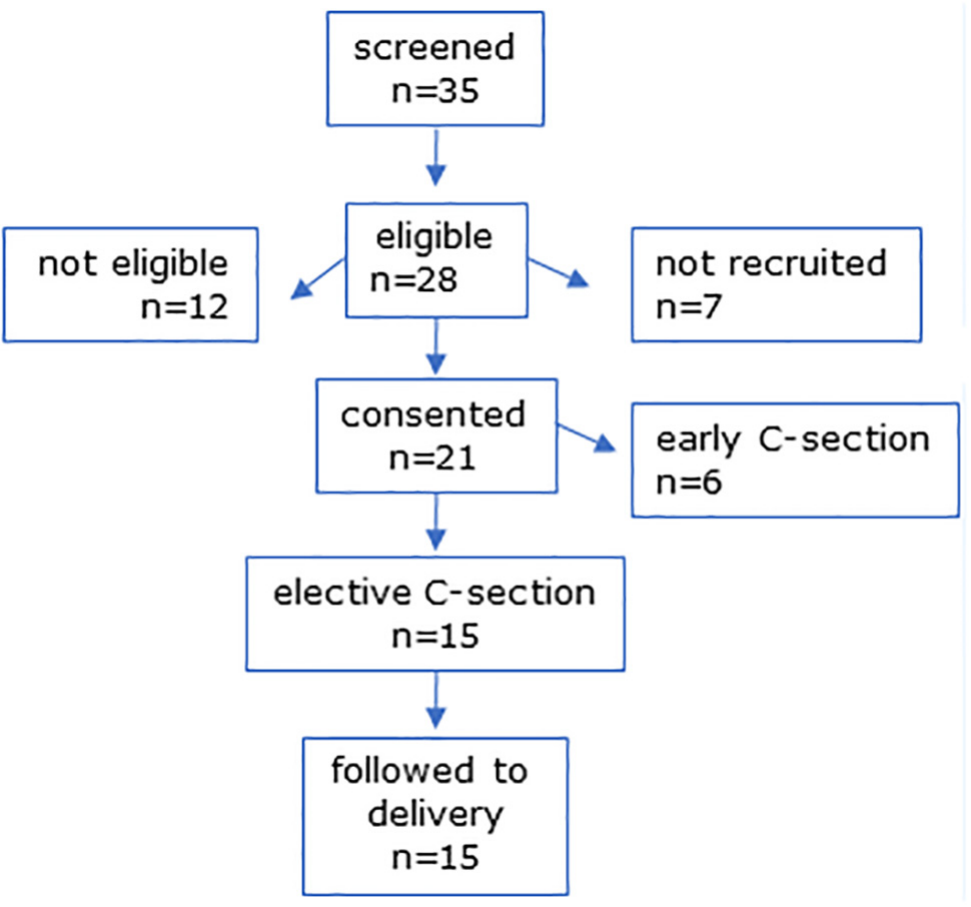
CONSORT diagram for MEL-P2 elective C-section participants.

The CONSORT diagrams show recruitment for all participants (**Figures 1** and **2**). Baseline characteristics of all participants are shown in **Table 1**. All groups were similar in age. Pregnant women had a slightly higher pre-pregnancy BMI than non-pregnant controls. **Figure 1** shows the CONSORT diagram for MEL-P. Approximately 120 women presented for their first scan and were screened for eligibility and willingness to talk to the researcher about the study; two subsequently refused consent and 30 were recruited. Two women withdrew consent before the second trimester and a further two withdrew before the third trimester, one due to identification of a fetal abnormality at further scan at 20 weeks and another due to pregnancy loss at 26 weeks. Twenty-six women were followed to the third trimester.

**Table 1:** Baseline characteristics of participants.

Group	Pregnant(n = 30)	Non-pregnant(n = 30)	Cesarean section(n = 15)
**Age** (years)	28 [17–37]	26 [21–44]	31 [21–40]
**BMI** (Kg/M^2^)	24.1 [18.8–38.5]	22.0 [19.5–31.9]	33.7 [24.9 –43.1]

Data shown as median [range]; BMI, body mass index.

Recruitment of the non-pregnant controls for MEL-P are shown in **Figure 1B**. Of the 44 women who initially expressed an interest, 35 were screened; 5 were ineligible due to chronic health conditions and/or medication use other than oral contraceptives, and 30 were recruited.

In MEL-P2, 35 women scheduled for CS were screened and 28 were eligible (**Figure 2**). Six were not eligible due to taking medication for chronic health issues (n = 6), 7 were not recruited due to incompatible CS dates, and one declined to take part. Six women were lost to follow-up due to CS prior to the scheduled date and 15 women were followed to delivery.

### Clinical Outcomes

All pregnancies followed to delivery resulted in a term livebirth. Nine of the women recruited in their first trimester (MEL-P) had spontaneous vaginal deliveries, 12 were induced, and 5 had elective CS, with no emergency CS or preterm deliveries. None of the women had pre-eclampsia and only one developed gestational diabetes. Of the women recruited who were scheduled for elective CS (MEL-P2), two had gestational diabetes (one taking metformin and other controlled by diet only), two had asthma with no exacerbations, and one had a congenital single kidney with no clinical symptoms of chronic kidney disease or hypertension. The reasons for CS were: previous CS (n = 6), breech presentation (n = 3), previous third degree tear (n = 2), placenta previa (n = 2), genital herpes (n = 1), and maternal request (n = 1).

### Melatonin and 6-Hydroxymelatonin Sulfate

**Figure 3:**
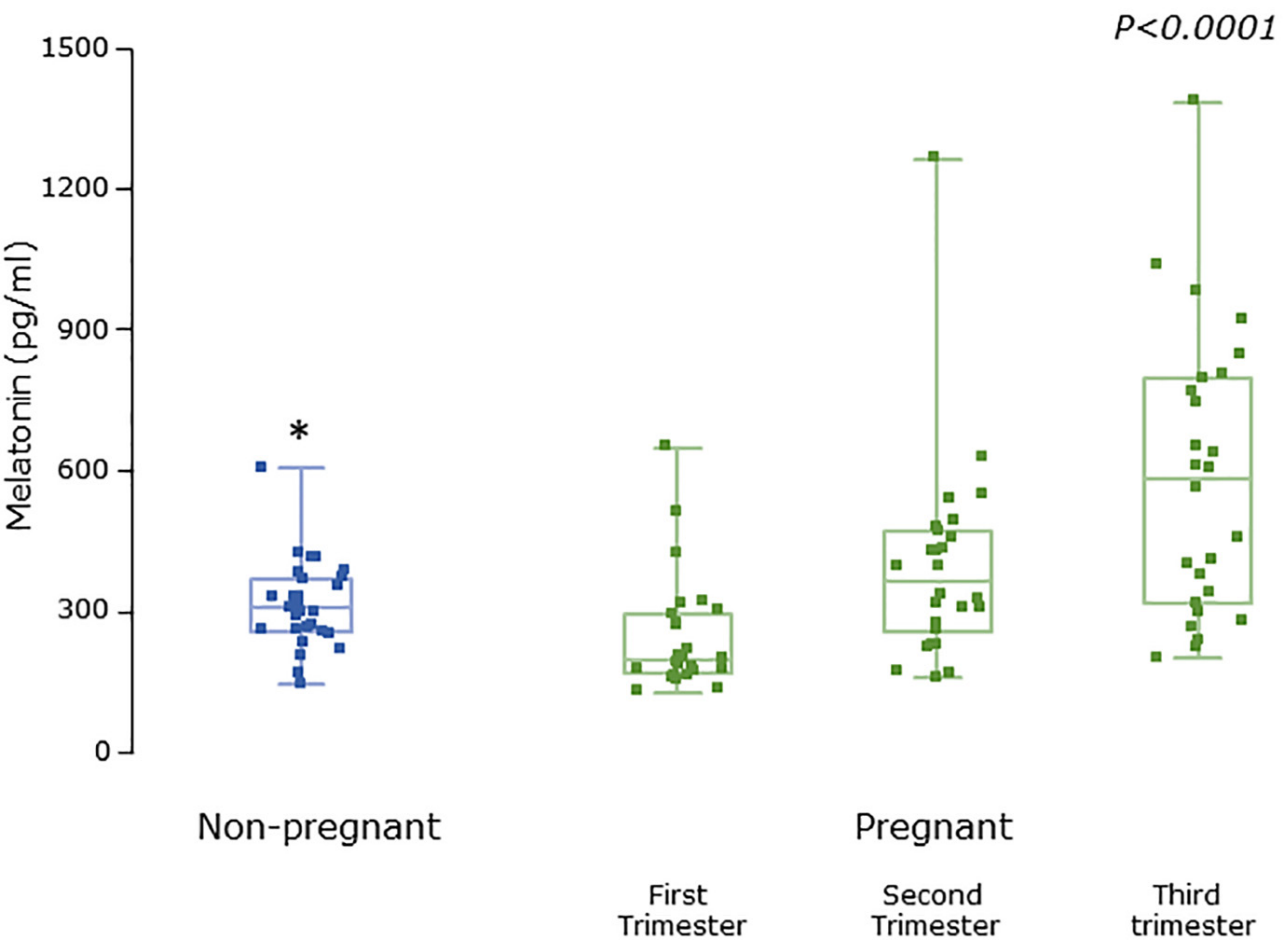
Serum melatonin levels from MEL-P in non-pregnant healthy control subjects (n = 30) compared to pregnant subjects throughout pregnancy (n = 26), *, significantly different from pregnant women in the first (P = 0.0025), second (P = 0.04), and third (P=0.0005) trimesters, Wilcoxon-Mann Whitney test. P value shown is Friedman test over the trimesters showing increasing melatonin levels (P < 0.0001). Data are shown as median, interquartile, and full range with individual raw data points overlaid.

**Figure 4:**
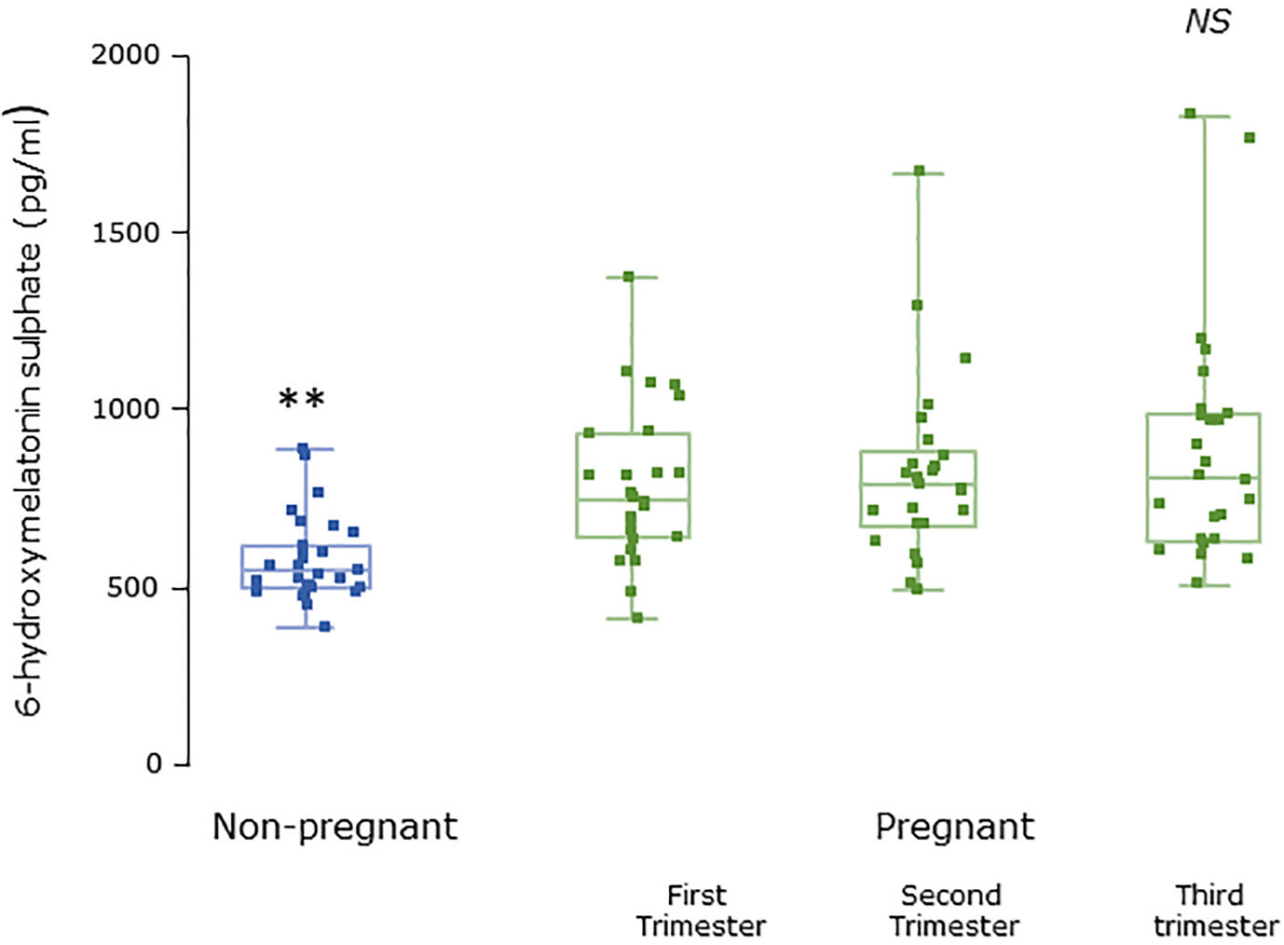
Serum 6-hydroxymelatonin sulfate levels from MEL-P in non-pregnant healthy control subjects (n = 30) compared to pregnant subjects throughout pregnancy (n = 26). **, significantly different from pregnant women in the first, second, and third trimesters (all P < 0.0001, Wilcoxon-Mann Whitney test). Data are shown as median, interquartile, and full range. NS, non significant.

**Figure 5:**
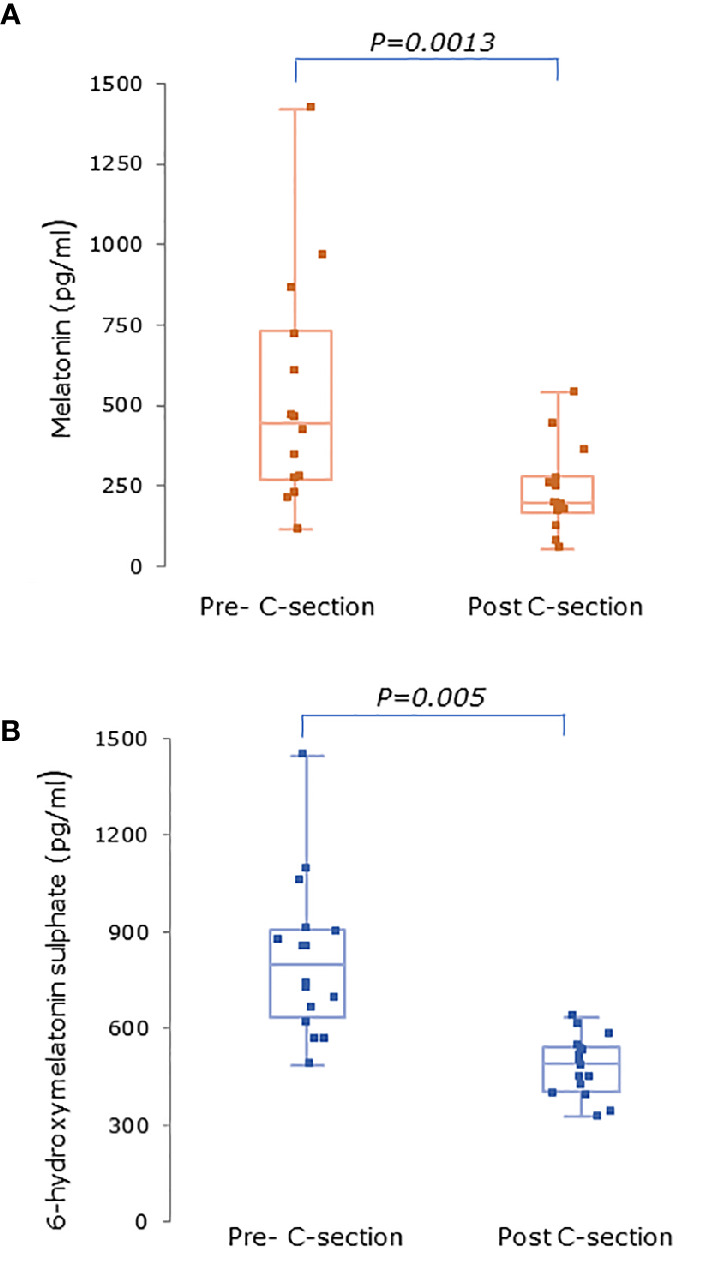
**(A)** Serum melatonin and **(B)** serum 6-hydroxymelatonin sulfate levels in pregnant women (n = 15) 3–6 days before and 24 h after elective C-section (MEL-P2). Levels were significantly after C-section (P = 0.0013 and P = 0.005 respectively, Wilcoxon-Mann Whitney test). Data are shown as median, interquartile, and full range.

**Figure 6:**
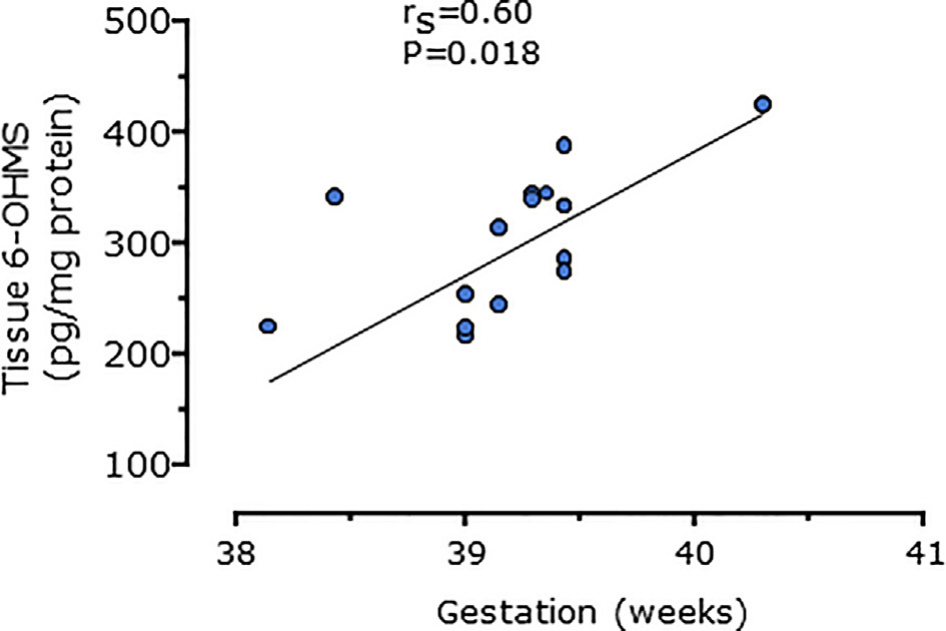
Relationship between placental 6-hydroxymelatonin sulfate levels after elective C-section and gestational age in weeks (n = 15, P = 0.016, Spearman’s test), from MEL-P2.

Serum concentrations of melatonin were variable between individuals but were significantly higher in all trimesters in pregnant women than the non-pregnant subjects (**Figure 3**, P = 0.025, P = 0.04, and P = 0.0005 respectively). Levels of melatonin increased significantly as pregnancy progressed, with levels in the third trimester being around 3-fold higher than in the first trimester (**Figure 3**, P < 0.0001). Serum levels of 6-OHMS were also variable and were significantly higher in pregnant women in all trimesters than in non-pregnant controls (**Figure 4**, all P < 0.0001) although there was no significant increase over pregnancy (**Figure 4**). We then went on to recruit another cohort of women scheduled for CS to determine what happened to melatonin levels once the pregnancy ended and to obtain placental tissue. Levels of maternal serum melatonin and 6-OHMS at 3–6 days before CS in the MEL-P2 cohort were similar to the levels seen in the third trimester in the MEL-P patient cohort. Twenty-four hours after CS, serum levels of both melatonin and 6-OHMS were markedly decreased compared to before delivery (P = 0.0013 and P = 0.005 respectively, **Figure 5**). Both serum melatonin and 6-OHMS levels 24 h after CS were slightly but significantly lower than in the non-pregnant controls (P = 0.046 and P = 0.0025 respectively). The median (range) level of 6-OHMS in placental tissue was 281.5 (217.1–346.8) pg/mg protein, which correlated with gestational date in weeks (r_s_ = 0.60, p = 0.018, **Figure 6**). Data are summarized in **Table 2**.

**Table 2:** Data summary.

MEL-P	Melatonin (pg/ml)	P values	6-OHMS (pg/ml)	P values
**1st trimester** (n = 26)	144.9 [76.5–611.4]	P < 0.0001	782.3 [599.1–1,408.5]	P = 0.17
**2nd trimester** (n = 26)	341.6 [102.1–1,246.4]		822.20 [254.3–1,705.2]	
**3rd trimester** (n = 26)	561.5 [185.3–1,372.5]		837.4 [538.8–1,800.5]	
**Controls** (n = 30)	272.9 [108.2–577.7]	P = 0.0025, P = 0.04, and P = 0.0005 compared to 1^st^, 2^nd^, and 3^rd^ trimesters	571.9 [404.6–903.1]	P < 0.0001 compared to all trimesters
**MEL-P2**				
**Before CS** (n = 15)	442.6 [115.3–1,422.7]	P = 0.0013	798.0 [564.4–1,447.9]	P = 0.005
**After CS** (n = 15)	196.4 [56.4–540.8]		493.0 [326.1–636.9]	

Median [range]; 6-OHMS, 6-hydtroxymelatonin sulfate; CS, cesarean section.

## Discussion

Our findings show that during normal pregnancy, circulating maternal serum levels of melatonin and 6-OHMS, the major metabolite of melatonin, increased markedly as pregnancy progressed and were significantly higher than in a non-pregnant cohort of similar age, even in the first trimester. In a separate cohort of women undergoing elective CS, maternal serum melatonin 3–6 days before delivery were also high and decreased considerably immediately after delivery. High levels of 6-OHMS were also seen in placental tissue which correlated with gestational age. These data show that melatonin production and/or metabolism is markedly increased during normal pregnancy. Furthermore, the levels of 6-OHMS found within placental tissue and their relationship with gestational age and as well as the sharp decrease in maternal serum melatonin and 6-OHMS after delivery suggests that the placenta is the source of the increased maternal melatonin.

Both exogenously administered and endogenously produced melatonin is rapidly metabolized, mainly in the liver, where it is mostly hydroxylated by the cytochrome P450 enzymes CYP1A1 and CYP1A2 into 6-hydroxymelatonin, with a small proportion de-methylated into N-acetylserotonin, along with limited hydroxylation outside the liver by CYP1B1 (16). These metabolites are either sulfated or glucoronidated, followed by urinary excretion, with 6-OHMS accounting for about 80% of the metabolic products (17, 18). Excretion levels of 6-OHMS in urine are highly variable between individuals (19, 20) but levels in serum are less so (20, 21). Levels of both melatonin and 6-OHMS can be quantified by enzyme immunoassay; the assays used here were highly specific, sensitive, and reproducible competitive enzyme linked immunosorbent assays (ELISA). We found here that melatonin and 6-OHM levels even in the non-pregnant controls were higher than in previous reports. We are unclear of the reason for this except that the method used here was an immunoassay rather than a chemical assay such as HPLC which is more generally used. Our results were consistent according to our precision analysis and the manufacturers report no cross reactivity between melatonin and similar compounds. We have used the same methods for other samples, for example after exogenous administration of melatonin to volunteers where we found that levels were related to dose (20). This has no impact on interpretation of our results. The high levels of melatonin and 6-OHMS in pregnant women could in theory represent either increased melatonin production or increased metabolism; increased production is more likely, since although metabolism of melatonin to 6-OHMS can be modulated by substances which affect enzymes involved in either hydroxylation or sulfation, these effects would decrease or delay metabolism; there are no reports of increased melatonin metabolism.

Previous studies from several years ago have reported melatonin levels during pregnancy in maternal, fetal and umbilical cord blood, with some conflicting results (6–8). Melatonin concentration in serum from different groups of pregnant women and from the umbilical cord immediately after birth was measured using radioimmunoassay in these studies. No statistically significant differences in daytime levels were seen but nighttime levels were significantly higher than in non-pregnant women only after 24 weeks gestation (8). Kivela (6) reported higher melatonin levels in late compared to early pregnancy in different cohorts of women. Analyzing samples from the same participants as pregnancy progresses is important since there is such marked inter-individual variation in metabolism due to the highly polymorphic nature of cytochrome P450 enzymes (17, 20, 22). We found significantly higher levels of melatonin and its major metabolite 6-OHMS compared to non-pregnant controls, which increased as pregnancy progressed and was coupled with marked decreases after removal of the placenta. This suggests that melatonin production in pregnancy is higher than non-pregnant women and that the placenta appears to be the source of increased circulating melatonin.

Our findings suggest that the placenta is the source of increased circulating melatonin in pregnancy. However sources from the mother and fetus as well as the placenta may also contribute. Given the anti-inflammatory effects of melatonin it is interesting to speculate that the high melatonin levels may be involved in the resistance of pregnant women to COVID-19 (23). Unlike in other studies (6, 8), the same patients were sampled throughout pregnancy which both increased the power of the study and minimized the contribution of inter-individual variation. Our participants included only those with singleton pregnancies; twin pregnancies are at higher risk of placental dysfunction and specifically pre-eclampsia and melatonin may be higher in twin pregnancies compared to singleton (8).

It was previously believed that endogenous melatonin from the pineal gland was solely responsible for circulating melatonin levels, but melatonin is present in most biological fluids and the enzymes responsible for its synthesis have been identified in the skin, immune cells, and the gastrointestinal tract (13, 14). Relevant here are the ovary and placenta (10–12) which have been reported to contain the enzymes needed for melatonin synthesis. Melatonin synthesis by placental cells is assumed although neither melatonin nor its metabolite levels have been previously reported; our data uphold the suggestion that the placenta is the source of high circulating maternal melatonin levels. We can find no previous reports of measurement of melatonin levels in human placenta apart from a study where melatonin was detected in placentas that had been processed for human consumption (24).

We found that maternal melatonin and 6-OHMS levels decreased markedly after the pregnancy ended, 24 h after CS (MEL-P2). A previous study some years ago also described lower levels after delivery (7). It was previously reported that umbilical cord blood melatonin concentrations differ in women giving birth *via* spontaneous vaginal delivery compared to CS (22, 25). It is not known what happens to melatonin levels during labor; prolonged high levels may take longer to return to non-pregnant levels than in women having CS. Melatonin has both antioxidant and anti-inflammatory actions (2, 3). Cytokine responses in women having CS are variable (22, 26) depending on circumstances yet oxidative stress was lower and antioxidant profile was augmented during CS delivery compared to spontaneous vaginal delivery (27). In non-pregnant patients undergoing major abdominal surgery, circulating melatonin levels decrease but return to pre-operative values within 2 days (28, 29), attributed to transient tumor necrosis factor α -mediated suppression of gene expression of the melatonin synthesizing enzymes. However, this is unlikely to explain the marked fall in melatonin levels we found after delivery and a more robust explanation is removal of the placenta. This area requires further research.

The physiological function of the elevated placental melatonin production remains unclear, but it could be speculated that it may be related to preparation for parturition and circadian regulation of melatonin receptor expression in human myometrium has been reported (30). Human parturition at term more commonly occurs late at night or early in the morning whereas preterm labor shows no such diurnal variation (29, 30). Our results coincide with the theory that melatonin may be involved in instigation of labor with significantly higher levels into the third trimester (15). This could have implications for induction of labor and preterm labor etiology; the role of melatonin in labor require further investigation.

Given the increased levels of melatonin and 6-OHMS compared with non-pregnant controls, even in the first trimester, melatonin may have a role in maintaining normal pregnancy. Of key clinical interest is whether low levels of melatonin may have a role in diseases of pregnancy. Administration of exogenous melatonin appears free of side effects in healthy non-pregnant subjects (20, 31) and an open label phase I single arm trial of exogenous melatonin in women with pre-eclampsia reported that melatonin was safe for mothers and fetuses (5). Exactly how melatonin may be involved in the pathophysiology of pregnancy is vital to before moving on to ascertain the potential for exogenous melatonin as a therapy to prevent adverse obstetric outcomes.

In summary we report here marked increases in daytime levels of melatonin and its major metabolite of melatonin in normal pregnancy, which decreased markedly after delivery, identifying the placenta as the potential source of the high circulating maternal melatonin levels. Increasing our understanding of the role of melatonin in the physiology and pathophysiology of pregnancy is vital, and confirmation of the role of the placenta in melatonin production may lead to the use of melatonin as a preventative treatment for obstetric conditions.

## Data Availability Statement

The raw data supporting the conclusions of this article will be made available by the authors, without undue reservation.

## Ethics Statement

The studies involving human participants were reviewed and approved by East Midlands, Nottingham 1 Research Ethics Committee for MEL-P, ref 16/EM/0412, and London-Bromley Research Ethics Committee for MEL-P2, ref 18/LO/0616. The patients/participants provided their written informed consent to participate in this study.

## Author Contributions

HG was the Chief Investigator and drafted the manuscript. HG and AW conceived and designed the studies and contributed to the data analysis and interpretation. HE and JF had equal responsibility for participant recruitment, sample collection, and analysis. BT had oversight of the sample analysis and data acquisition. All authors contributed to the article and approved the submitted version. We confirm that all authors:

1. Have made substantial contributions to conception and design, or acquisition of data, or analysis and interpretation of data;
2. Been involved in drafting the manuscript or revising it critically for important intellectual content;
3. Given final approval of the version to be published. Each author should have participated sufficiently in the work to take public responsibility for appropriate portions of the content; and
4. Agreed to be accountable for all aspects of the work in ensuring that questions related to the accuracy or integrity of any part of the work are appropriately investigated and resolved.
5. All authors agree on the order in which their names are listed in the manuscript.

## Funding

The work was funded by non-specific institutional sources.

## Conflict of Interest

The authors declare that the research was conducted in the absence of any commercial or financial relationships that could be construed as a potential conflict of interest.
